# *Toxoplasma gondii* seroprevalence in breeding pigs in Estonia

**DOI:** 10.1186/s13028-017-0349-1

**Published:** 2017-12-11

**Authors:** Azzurra Santoro, Maarja Tagel, Kärt Must, Miia Laine, Brian Lassen, Pikka Jokelainen

**Affiliations:** 1 Department of Veterinary Medicine, University of Perugia, Via San Costanzo 4, 06126 Perugia, Italy; 2 Institute of Veterinary Medicine and Animal Sciences, Estonian University of Life Sciences, Kreutzwaldi 62, 51014 Tartu, Estonia; 3 Department of Veterinary Disease Biology, Faculty of Health and Medical Sciences, University of Copenhagen, Grønnegårdsvej 15, 1870 Frederiksberg C, Denmark; 4 Faculty of Veterinary Medicine, University of Helsinki, P.O. Box 66, 00014 Helsinki, Finland; 5 Statens Serum Institut, Artillerivej 5, 2300 Copenhagen S, Denmark

**Keywords:** Breeding herd, Epidemiology, Porcine, Serology, *Sus scrofa domesticus*, Swine, Toxoplasmosis, Zoonosis

## Abstract

**Background:**

*Toxoplasma gondii* is a widespread occurring parasite infecting warm-blooded animals, including pigs and humans. The aims of this study were to estimate the prevalence of anti-*T. gondii* antibodies and to evaluate risk factors for *T. gondii* seropositivity in breeding pigs raised in Estonia. Sera from 382 pigs were tested with a commercial direct agglutination test, using a cut-off titer of 40 for seropositivity, for the presence of anti-*T. gondii* immunoglobulin G antibodies.

**Results:**

Twenty-two (5.8%) of the 382 pigs tested seropositive for *T. gondii*, and 6 of the 14 herds had at least one seropositive pig. The proportion of seropositive pigs within the herds ranged between 0 and 43%. Gender appeared as a significant factor, with sows having 5.6 times higher odds to be seropositive to *T. gondii* than boars. Seroprevalence did not increase with age.

**Conclusions:**

Anti-*T. gondii* antibodies were present in a substantial proportion of breeding pig herds in Estonia. On the other hand, the presence of herds without seropositive pigs illustrates that porcine *T. gondii* infections can be avoided even in a country where the parasite is endemic and common in several other host species.

## Background

*Toxoplasma gondii* is a protozoan parasite with worldwide distribution. Recently, the Food and Agriculture Organization (FAO) and the World Health Organization (WHO) ranked it 4th among foodborne parasites causing the greatest global concern [1].

All warm-blooded animals, including humans and pigs, can host *T. gondii*. Both humans and pigs, being omnivores, can acquire the infection via ingestion of tissues of other hosts that carry the parasite, via ingestion of oocysts from a contaminated environment, or congenitally [2]. Infections with *T. gondii* can be subclinical, but toxoplasmosis can have severe consequences for both human and porcine health [3–5].

Pork is considered a major source of human *T. gondii* infections in Europe and the USA [2, 6]. Viable *T. gondii* parasites have been isolated from unprocessed tissues of infected pigs as well as from commercial cuts such as ham, bacon, and pork tenderloin [7, 8]. Possible sources of naturally-acquired porcine *T. gondii* infections have been investigated in studies evaluating different risk factors for porcine *T. gondii* infection [2, 4]. Some investigated risk factors, such as the age of the pigs and herd size, do not provide us with useful clues regarding the sources of infection, whereas others do. For example, the access of seropositive juvenile cats to areas where sows were housed [9], direct access of cats to pig feed [10], and a high density of cats at the farm [11] have been shown to be positively associated with *T. gondii* seropositivity in pigs, suggesting oocyst contamination of pig feed and the farm environment as possible sources of the infection. Inadequate rodent control has also been associated with *T. gondii* seropositivity in pigs, suggesting infected rodents as a possible source of *T. gondii* infection for pigs [12].

*Toxoplasma gondii* infection has been reported in humans and pigs worldwide [2, 4]. The European Food Safety Authority (EFSA) has listed toxoplasmosis among the diseases to be reported by European Union (EU) member states according to their epidemiological situation and emphasized the lack of representative data for *T. gondii* in humans, animals, and food [13]. Moreover, EFSA has included *T. gondii* among the most relevant biological hazards in the context of meat inspection of swine and has pointed out that the current meat inspection is unable to detect the parasite [14].

In Estonia, a recent nationwide study estimated that *T. gondii* seroprevalence was 55.8% in the human population in general, and 74.4% in a separate group of animal caretakers [15]. Further epidemiological data from other host species, including domestic cats [16], cattle [17], and wild boars [18], indicate that *T. gondii* is endemic also in these populations, and present also in the environment. Based on a EU report from 2013, none of 20 pigs tested from Estonia were *T. gondii* seropositive [19]. While the consumption of pork has increased from 26.8 kg per person in 2002 to 44.2 kg per person in 2016 [20], there have been no studies with larger sample size on prevalence of subclinical *T. gondii* infection in domestic pigs in Estonia. Moreover, there are no reports of clinical porcine toxoplasmosis from Estonia.

The aims of our cross-sectional seroepidemiological study were to estimate *T. gondii* seroprevalence and to evaluate potential risk factors for *T. gondii* seropositivity in breeding pigs in Estonia. More specifically, we estimated the animal-level prevalence of anti-*T. gondii* immunoglobulin G (IgG) antibodies and evaluated both animal-level and farm-level risk factors for animal-level *T. gondii* seropositivity.

## Methods

### Study population and study design

The samples investigated were originally collected for national surveillance of other infectious diseases. The surplus of them were used in two other studies [21, 22] in addition to our study. Serology was performed blinded, and the data were coded and treated confidentially.

At the end of 2011, there were 365,700 pigs in Estonia [23]. This included 30 breeding herds with 15,337 animals, including boars used for insemination [24].

In this cross-sectional seroepidemiological study, we analyzed blood samples from breeding pigs in Estonia for evidence of naturally acquired *T. gondii* infections. The sample was a convenience sample.

### Samples

**Fig. 1:**
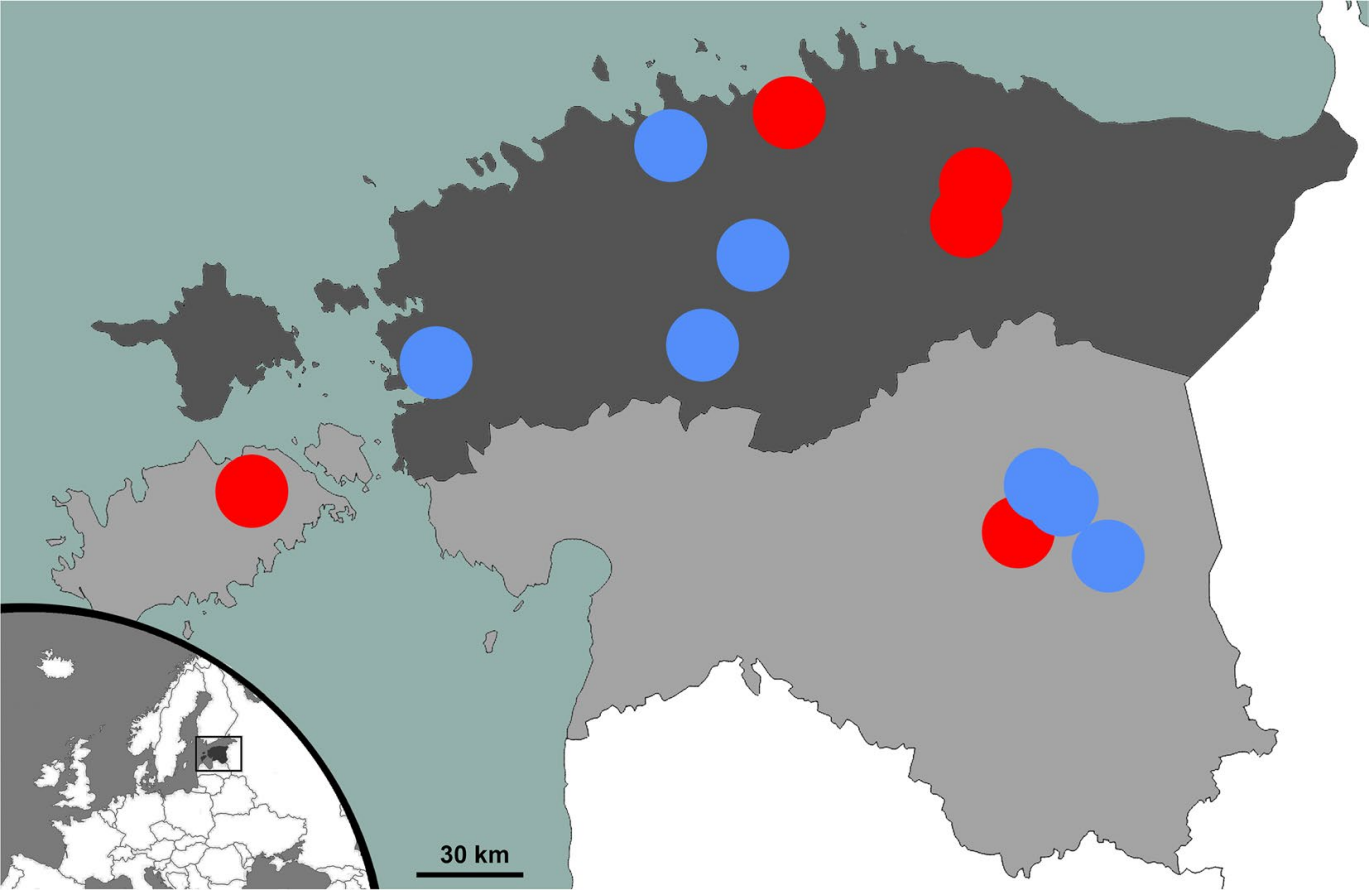
A map of Estonia showing the geographical location of the farms included in this study. From each herd, 5–52 breeding pigs were tested for specific antibodies against *Toxoplasma gondii*. The herds with at least one *T. gondii* seropositive pig are marked with red dots and the herds with no *T. gondii* seropositive breeding pigs are marked with blue dots. The northern counties are shown with dark grey color, and the southern counties are shown with light grey color. The exact location of one herd with at least one *T. gondii* seropositive breeding pig and one herd with no *T. gondii* seropositive breeding pigs, both located in the southern part of the country, were unknown

The samples available for our study were sera from 382 domestic pigs from 14 breeding herds located in seven of the 15 counties in Estonia (Fig. 1). Information was not available for how the herds had been selected, but random sampling had been used at the animal-level. The number of pigs that had been sampled per herd was 5–52. The blood samples had been collected in 2012 by veterinarians of the Estonian Veterinary and Food Board. The samples had been collected on the farms, from the jugular vein of live pigs, into vacuum tubes without reagents. The sera had been separated by centrifugation and were stored at − 20 °C until analysis.

The sample size of 382 domestic pigs was evaluated to be sufficient for estimating the animal-level seroprevalence using the Epi Tools software calculator [25], with an expected seroprevalence range of 3.2–4.2% [26, 27], confidence level of 95%, and population size of 15,337 [24]. Ignoring clustering, the minimum required sample size would have been 48–62 samples. Clustering was expected, thus we did not settle for the minimum required sample size and included all the 382 samples that were available.

### Background information

Background information for each pig was extracted from the registers of the Estonian Livestock Performance Recording Ltd and included gender, breed, and age in days and in years. Gender was unknown for 58 pigs, breed for 87 pigs, and age for 71 pigs. For each farm, we had information about herd size and farm location.

The sample consisted of breeding sows (66%) and boars (34%) (Table 1). Pigs that were crossbred Estonian Landrace breed and Estonian Large White breed were most common (36%), followed by pure Estonian Landrace breed (33%) and pure Estonian Large White breed (18%) (Table 1). The age of the pigs ranged from 178 to 2742 days (mean 726 days, median 651 days); that is, from 6 months to 7 years (mean 1.99 years, median 1.78 years). The herd size range was 58–11,295 (mean 3786, median 2366) pigs. Seven of the farms were located in the northern part of the country and seven of the farms were located in the southern part of the country (Fig. 1; divided as in [18]).

**Table 1:** Animal-level *Toxoplasma gondii* seroprevalence in breeding pigs in Estonia

	No. of pigs	No. of seropositive pigs	Seroprevalence (95% CI)
Gender^a^			
Sow	213	20	9.4** (6.0–13.9)
Boar	111	2	1.8** (0.3–5.8)
Breed^a^			
Landrace/Large White	106	8	7.5 (3.6–13.8)
Landrace	98	10	10.2* (5.3–17.4)
Large White	53	2	3.8 (0.6–11.9)
Other^b^	38	0	0.0* (0.0–7.6)
Age (year)^a^			
< 1	72	2	2.8 (0.5–8.9)
≥ 1	239	19	7.9 (5.0–11.9)
Herd size			
< 2000 pigs	170	6	3.5 (1.4–7.2)
≥ 2000 pigs	212	16	7.5 (4.5–11.7)
Location of the farm			
Northern part of the country	150	10	6.7 (3.4–11.6)
Southern part of the country	232	12	5.2 (2.8–8.6)
Total	382	22	5.8 (3.7–8.5)

*CI* confidence interval, Mid-P exact

* Significantly different seroprevalence (P < 0.05)

** Significantly different seroprevalence (P < 0.01)

^a^ Some information unknown for some animals

^b^ The other breeds were pure Piétrain, pure Duroc, Duroc/Landrace, Piétrain/Duroc, Piétrain/Landrace, and Piétrain/Landrace/Large White

### Serology

The serum samples were tested for the presence of specific IgG antibodies against *T. gondii* using a commercial direct agglutination test (Toxo-Screen DA, bioMérieux, Marcy-l’Étoile, France), according to the manufacturer’s instructions. In this method, possible immunoglobulin M antibodies are denatured by 2-mercaptoethanol.

The dilution of the samples was 1:40 and pigs testing positive were defined as seropositive; that is, the cut-off for seropositivity was titer of 40 [18]. Only samples yielding clear positive results (an agglutination yielding a mat that covered at least half of the bottom of the well) were interpreted as positive, whereas borderline reactions were interpreted as negative. All plates contained the positive and negative controls provided in the kits, in two dilutions (1:40 and 1:4000), and an antigen control, as instructed by the manufacturer. The antigen control, consisting of all the reagents of the kit (i.e. no serum), confirmed that autoagglutination did not occur.

### Statistical analyses

We estimated the seroprevalence (not adjusted, i.e. apparent seroprevalence) at animal-level and also report the proportion of herds that had at least one seropositive pig and the range of proportion of seropositive pigs within each herd. OpenEpi was used to calculate confidence intervals (CI, Mid-P exact) and to evaluate the differences using two by two Tables (2-tailed *P* values, Mid-P exact) [28]. Logistic regression analyses using Stata software 13.1 (StataCorp, College Station, TX, USA) were used to evaluate the potential risk factors. P values < 0.05 were considered statistically significant.

In the risk factor analyses, the outcome was a dichotomous animal-level outcome: each pig was either seronegative or seropositive. We evaluated five potential risk factors for seropositivity. The animal-level variables we evaluated were gender, breed and age. Breed was evaluated as a dichotomized variable: one group consisted of purebred Estonian Landrace pigs and crossbred Estonian Landrace pigs (Estonian Landrace breed crossed with Duroc, Estonian Large White, and Piétrain), while the other group consisted of pigs that had no Estonian Landrace breed in them (i.e. pigs that were purebred Piétrain, purebred Duroc, purebred Estonian Large White, or crosses of these three breeds). Age was evaluated in three ways: dichotomized as < 1 year vs. ≥ 1 year; as a continuous variable; and as different age categories based on the age distribution of the sample (dichotomized at 2 years, dichotomized at 3 years, categorized by every 3 months, categorized by every 6 months, categorized by every 12 months, categorized at quartiles, categorized at median, categorized at mean). The farm-level variables we evaluated were herd size (dichotomized: < 2000 vs. ≥ 2000 pigs) and location of the farm (dichotomized: the northern part of the country vs. the southern part of the country, as in [18]).

First, univariable logistic regression models were used to evaluate each potential risk factor separately. Multivariable logistic regression model building was then attempted by including all the variables, followed by backward elimination of those with *P* value ≥ 0.05 that did not act as confounders. We used > 20% change in the odds ratio as an indication of confounding. To account for clustering, the variable ‘farm’ was tried as a random factor in all the models.

## Results

### Seroprevalence

The overall *T. gondii* seroprevalence estimate in breeding pigs in Estonia was 5.8% (95% CI 3.7–8.5); 22 of 382 animals tested positive for anti-*T. gondii* IgG antibodies (Table 1). This estimate was not statistically significantly different from the earlier result of 20 pigs testing seronegative [19]. Six of the 14 herds (42.9%, 95% CI 19.6–68.9) had at least one seropositive pig. The proportion of seropositive pigs of pigs tested within each herd ranged between 0.00 and 42.9%, being ≥ 25% in two of the herds.

### Risk factors

**Fig. 2:**
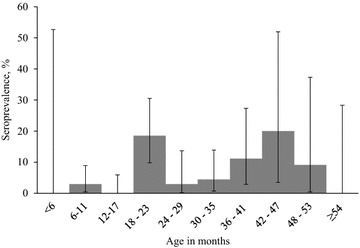
*Toxoplasma gondii* seroprevalence in breeding pigs in Estonia, by age in months

*Toxoplasma gondii* seroprevalence was significantly higher (P < 0.01) in sows than in boars (Table 1), and based on the univariable logistic regression model (without a random factor), sows had 5.65 (95% CI 1.3–24.6) times higher odds to test seropositive than boars. The seroprevalence was highest in the purebred Estonian Landrace pigs (Table 1). The two herds with ≥ 25% seropositive pigs of the pigs tested both included exclusively purebred or crossbred Estonian Landrace pigs. Age was not a significant factor in univariable analyses, neither as a dichotomized variable, as a continuous variable, nor as different age categories, and a histogram (Fig. 2) showed no obvious pattern for the seroprevalence by age. Seroprevalence in pigs from larger herds (≥ 2000 pigs) did not differ significantly from the seroprevalence in pigs from smaller herds (< 2000 pigs) (Table 1). Three of the herds that had at least one seropositive pig were located in the northern part of the country and three were located in the southern part of the country (Fig. 1). None of the plausible risk factors were significant variables when tried in a multivariable model nor when ‘farm’ was included as a random factor in the models.

## Discussion

In Europe, reports available show that animal-level *T. gondii* seroprevalence in domestic pigs ranges in the investigated regions from 0.4 to 64.4% [2, 29]; while looking at reports from northern European countries specifically, the range is from 3.2% in finishing pigs in Finland [26] to 33.7% in indoor sows in Denmark [30]. Seroprevalence estimates similar to the estimate obtained in breeding pigs in our study (5.8%) have been reported in pigs from Latvia (4.2%) [27] and Sweden (calculated from the numbers reported: 5.7%) [31].

It is important to note that, the estimates of *T. gondii* seroprevalence obtained in different studies are not directly comparable due to different study designs and methodology. The serology method used in our study to detect anti-*T. gondii* antibodies in breeding pigs is widely used [4, 32]. The seroprevalence in breeding pigs from Estonia did not differ significantly from estimates obtained using the same method and cut-off titer in pigs from Austria (6.5%) [33] and Portugal (7.1%) [34], but was significantly lower than *T. gondii* seroprevalence estimates obtained using the same method and cut-off in pigs from Canada (9.4%) [35] and Poland (26.4%) [36]. In Spain, *T. gondii* seroprevalence in pigs was 19.0% using 25 as the cut-off titer, while 5.8% tested positive when the cut-off titer was 50 (calculated from numbers reported) [37], which was similar to the seroprevalence estimate in breeding pigs in our study, obtained with 40 as the cut-off titer. Our study included pigs from breeding herds, whereas several of the other studies investigated samples collected from finisher pigs (i.e. pigs in the late stage of rearing, i.e. > 70 kg live weight). In Germany, the animal-level ELISA-based *T. gondii* seroprevalence estimate in breeding pigs was 18.5% [38], statistically significantly higher than our estimate from Estonia, while the proportion of herds with at least one seropositive animal, 69.1% [38], did not differ statistically from our result from Estonia.

*Toxoplasma gondii* seroprevalence differed significantly by gender, and sows had higher odds of testing seropositive than boars (univariable analysis). Possible explanations for this include differences in keeping sows and boars. For example, boars may be more likely kept in single pens and they may get different feed. If there are cats on the farm, they might be discouraged to approach the boars, and thus the immediate environment of the boars might be less likely to be contaminated with feline feces.

Some studies have shown an increase in *T. gondii* seroprevalence in pigs with age [39, 40], whereas others have not [41, 42]. No increase in *T. gondii* seroprevalence with age was observed in our study, despite the fact that we were able to evaluate a wide age range. Instead, we observed clustering of seropositive animals in specific age-groups (Fig. 2) and herds. A possible explanation for this observation could be focal feed- or waterborne outbreaks in the herds: a group of pigs of the same age on a farm were likely housed and fed together, and could have been given feed or water contaminated with infective *T. gondii*. Data on previous serostatus, direct detection of the parasites in feed or water, or genotyping the parasites could have confirmed the possible outbreaks.

Despite apparent differences in seroprevalence by pig breed (Table 1), being of the local Estonian Landrace breed did not appear as a risk factor for *T. gondii* seropositivity in further analyses. No statistical indication of herd size being associated with seropositivity was found, and no geographical pattern was evident. The latter result is in line with the quite even geographical distribution of seropositive wild boars in Estonia [18].

It should be noted that we used a single dilution of the samples and the titers were not evaluated. In addition, our cut-off titer for seropositivity of 40 can be considered high. Consequently, any pigs with low titers or recent infections (i.e. prior to antibody production), as well as undetected prozone phenomena (i.e. in case of too high antibody concentration for the test, few antibodies bind to more than one antigen and thus an agglutination mat is not formed, and a false negative result is seen), could contribute to an underestimation of the actual prevalence.

A concordance between *T. gondii* seropositivity and the presence of *T. gondii* in the tissues of pigs has been reported [43]. However, while serological screening is considered suitable for identifying high risk pig herds or individual pigs, serology is an indirect method and a negative result is not a guarantee of an uninfected pig or *T. gondii*-safe meat [43, 44]. All edible parts of a pig infected with *T. gondii* should be considered infectious since little variation in parasite load has been found between different skeletal muscles [44]. In Estonia, the amount of pork eaten per person has increased from 33.7 kg in 2012 to 41.8 kg in 2015 [20]. One single pig is estimated to be consumed by 300–400 people [45], thus even a low *T. gondii* prevalence must be considered an indication of food safety risk. However, meat from breeding pigs is generally processed in ways capable of inactivating viable stages of *T. gondii* [4]. Based on this, it has been suggested that meat from infected breeding pigs would be a minor source of infection in comparison with meat from infected finisher pigs [46]. A source attribution study could evaluate whether these assumptions are also true in Estonia.

Eating raw or undercooked meat of infected animals and eating food cross-contaminated with meat from infected animals are potential food safety risks not only for humans, but also for other host species, including cats. Our recent studies from Estonia and Finland revealed that a considerable proportion of domestic cats receive raw meat [16, 47, 48].

Both *T. gondii* and another tissue-dwelling zoonotic parasite group *Trichinella* spp. are endemic and common in Estonia in several of their host species; for example, the *T. gondii* seroprevalence was 24.0%, and the *Trichinella* seroprevalence was 42.1% in free-ranging wild boars [18, 22]. The *T. gondii* seroprevalence estimate in free-ranging wild boars [18] was obtained using the same serology method and cut-off that we used in this study, and it was significantly higher than our seroprevalence estimate in domestic pigs (24.0% vs. 5.8%; P < 0.01). This difference could be interpreted to exemplify the partial effectiveness of protecting Suidae from *T. gondii* by keeping pigs on farms, compared with free-ranging. Furthermore, *T. gondii* seropositive pigs could be seen as an indicator of unsuccessful biosafety measures on the farms—they have ingested tissues of infected animals or something contaminated with feces of infected felids that shed oocysts. Unfortunately, we did not have detailed information on the management and biosafety measures applied on the farms included in this study. Nevertheless, it should be noted that 374 of the pigs included in this study, including the 22 *T. gondii* seropositive pigs, were tested for antibodies against *Trichinella* spp. in our other study [22], and all tested negative. As the infection pressure of both parasites is high in Estonia, these results suggest that biosafety measures sufficient to stop *Trichinella* spp. infections are insufficient against *T. gondii*. *Trichinella* spp. infection can be acquired by carnivorism only, and the biosafety measures against *Trichinella* spp. infections target that. *Toxoplasma gondii* infection can be acquired by carnivorism or from oocysts, and thus biosafety measures targeting carnivorism only are insufficient. The EU-regulation on official control for *Trichinella* spp. infections in pigs focuses on carnivorism: the requirements for a farm to be recognized as applying controlled housing conditions include preventing mammals and carnivorous birds from having access to the buildings where pigs are kept, not allowing pigs access outdoors without specific risk analysis, and storing feed in closed containers that are impenetrable to rodents [49]. A previous national legal document on preventing *Trichinella* spp. infections in pigs listed e.g. regular rodent control, destroying of dead animals, and heat-treatment of animal-derived feed, while avoiding cats on the farms was also specifically mentioned [50]. While these measures that are targeted against *Trichinella* spp. infections can be expected to also have effect against *T. gondii*, they are not sufficient to avoid *T. gondii* infections: the prevention of oocyst-contamination, in particular of feed and water, has not been emphasized. A targeted *Toxoplasma*-control in domestic pigs would protect humans and other hosts that eat pork [2, 6], and porcine health and welfare [4].

## Conclusions

Our results provide evidence that *T. gondii* infection was present in Estonian breeding pig herds, while being of the local Estonian Landrace breed did not appear to be a risk factor for *T. gondii* seropositivity. Sows had higher odds to test seropositive than boars, which could be due to their different management. The clustering of seropositive pigs in specific age groups and herds could be indicative of point sources of infection that were shared by the groups of pigs in the herds.
